# Acerogenin A from *Acer nikoense* Maxim Prevents Oxidative Stress-Induced Neuronal Cell Death through Nrf2-Mediated Heme Oxygenase-1 Expression in Mouse Hippocampal HT22 Cell Line

**DOI:** 10.3390/molecules200712545

**Published:** 2015-07-09

**Authors:** Dong-Sung Lee, Byung-Yoon Cha, Je-Tae Woo, Youn-Chul Kim, Jun-Hyeog Jang

**Affiliations:** 1 Department of Biomedical Chemistry, College of Health and Biomedical Science, Konkuk University, Chung-Ju 380-701, Korea; E-Mail: dslee@kku.ac.kr; 2 Research Institute for Biological Functions, Chubu University, 1200 Matsumoto, Kasugai, Aichi 487-8501, Japan; E-Mails: bycha@isc.chubu.ac.jp (B.-Y.C.); jwoo@isc.chubu.ac.jp (J.-T.W.); 3 College of Pharmacy, Wonkwang University, Iksan 570-749, Korea; E-Mail: yckim@wku.ac.kr; 4 Department of Biochemistry, Inha University School of Medicine, Incheon 400-712, Korea

**Keywords:** acerogenin A, mouse hippocampal HT22 cells, neuro-protective effects, heme oxygenase-1, nuclear factor-E2-related factor 2

## Abstract

Oxidative cell damage contributes to neuronal degeneration in many central nervous system (CNS) diseases such as Parkinson’s disease, Alzheimer’s disease, and ischemia. Inducible heme oxygenase (HO)-1 acts against oxidants that are thought to play a key role in the pathogenesis of neuronal diseases. The stem bark of *Acer nikoense* Maxim (Aceraceae) is indigenous to Japan; it has been used in folk medicine as a treatment of hepatic disorders and eye diseases. Acerogenin A, a natural compound isolated from Japanese folk medicine *A. nikoense*, showed neuroprotective effects and reactive oxygen species (ROS) reduction on glutamate-induced neurotoxicity by inducing the expression of HO-1 in mouse hippocampal HT22 cells. Furthermore, acerogenin A caused the nuclear accumulation of nuclear factor-E2-related factor 2 (Nrf2) and the activation of the PI3K/AKT signaling pathways. In this study, we demonstrated that acerogenin A effectively prevents glutamate-induced oxidative damage, and HO-1 induction via PI3K/Akt and Nrf2 pathways appears to play a key role in the protection of HT22 cells. Therefore, this study implies that the Nrf2/HO-1 pathway represents a biological target and that acerogenin A might be a candidate for the prevention of neurodegeneration.

## 1. Introduction

The initial factors that cause neuronal death remain unclear. Brain tissues are vulnerable to oxidative stress and inflammation that may occur physiologically as a component of aging process or pathologically as a result of neurodegenerative disease [1]. Oxidative stress not only results in accidental damage to cells, but also actively triggers intracellular signaling pathways that lead to cell death [2]. Many neurodegenerative diseases such as ALS, Parkinson’s, Alzheimer’s, and Huntington’s occur as a result of neurodegenerative processes. Neuronal oxidative stress has been postulated as the underlying basis for neuronal cell death in neurodegenerative diseases [3]. To counteract oxidative stress, cells possess a variety of antioxidant systems. There are enzymatic antioxidants, such as glutathione peroxidase, superoxide dismutase, catalase, and phase II detoxifying enzymes, which contribute to xenobiotic detoxification and expression of stress response proteins, such as heme oxygenase-1 (HO-1) [4]. HO-1 is a protein that plays a cytoprotective role against neuronal apoptosis and neuro-inflammation [5]. HO-1 is an enzyme that catalyzes the degradation of the heme group to produces carbon monoxide (CO), biliverdin, and free iron. HO-1 and its enzymatic by-products appear to play important roles in regulating biological responses, including oxidative stress, inflammation, and cell proliferation [6]. The expression of HO-1 also has cytoprotective effects against glutamate-induced oxidative cytotoxicity in HT22 cells [7,8].

The stem bark of *Acer nikoense* Maxim (Aceraceae) is indigenous to Japan which has been used in folk medicine as a treatment of hepatic disorders and eye diseases [9]. Constituents previously isolated from *A. nikoense* are diarylheptanoids, phenolic compounds, and tannin, and their bioactivities such as anti-inflammatory effects and protective effects against hepatic injury were also reported [10,11,12]. Acerogenin A was isolated from *A. nikoense*. Many products structurally related to acerogenin A have been identified. This compound exerts several biological effects such as the inhibitory effects on degranulation in basophilic leukemia cells [13], the protective effects against hepatic injury [14], and the inhibitory effects on nitric oxide production in lipopolysaccharide-activated macrophages [11], which is a specific activity of acerogenin A. However, there have been no studies on the molecular targets of acerogenin A and the mechanisms underlying their anti-neurodegenerative biological activities. In the present study, we isolated acerogenin A and investigated its neuroprotective effects on glutamate-induced oxidative toxicity in mouse hippocampal HT22 cells through nuclear factor E2-related factor 2 (Nrf2)-dependent HO-1 expression via activation of the phosphatidylinositol 3-kinase (PI3K)/AKT pathways.

## 2. Results and Discussion

### 2.1. Effects of Acerogenin A on Glutamate-Induced Cytotoxicity and Inhibition of ROS Generation in HT22 Cells

Neurodegenerative diseases are incurable, and these results in progressive degeneration or damage of various neuronal cells. Neuronal oxidative stress has been postulated to be the underlying basis for neuronal cell death in neurodegenerative diseases [1,2]. Natural products and traditional medicines afford significant promise for the identification of bioactive components and their development into drugs for the treatment of various human disorders, including neuronal diseases. Acerogenin A was isolated from the stem bark of *Acer nikoense* Maxim, and it has been used in folk medicine as a remedy for hepatic disorders and eyewash. Nagai *et al*. first isolated acerogenin A from the stem bark of *A. nikoense* in 1976 [15]. In addition, other studies showed that acerogenin A exert several biological actions [11,13,14], but no study has yet been published on the mechanism studies of neuro-protective active components from *A. nikoense* or acerogenin A

**Figure 1:**
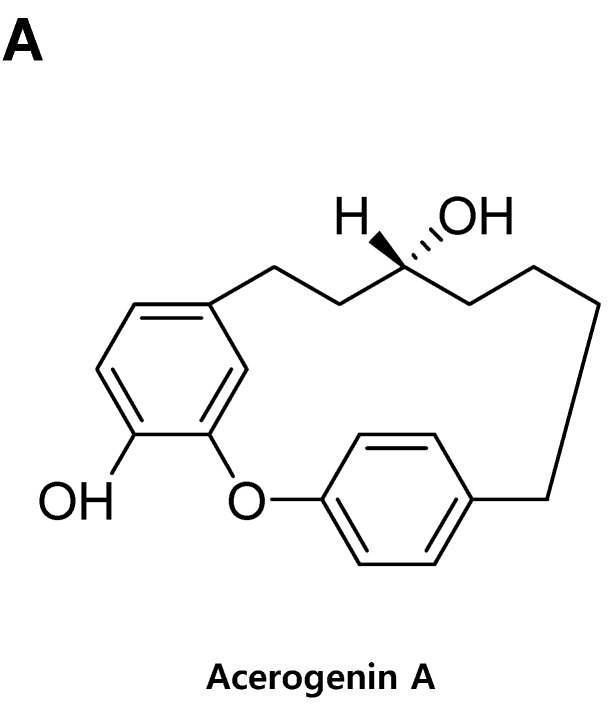

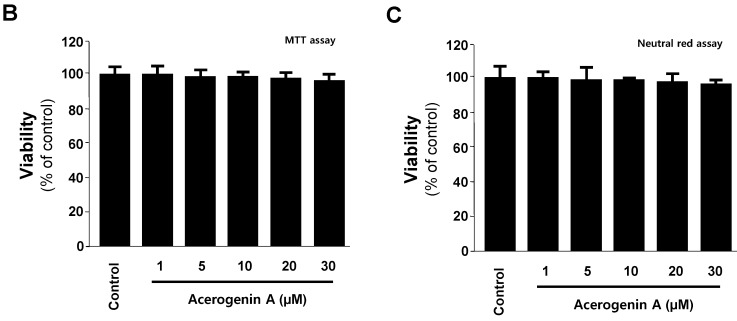
The structure of acerogenin A (**A**) and the effects of acerogenin A on cell viability by MTT assay (**B**) and neutral red assay (**C**). HT22 cells were incubated for 48 h with various concentrations of acerogenin A (1–30 μM) (**B**,**C**). Data are presented as mean ± SD values of three independent experiments.

**Figure 2:**
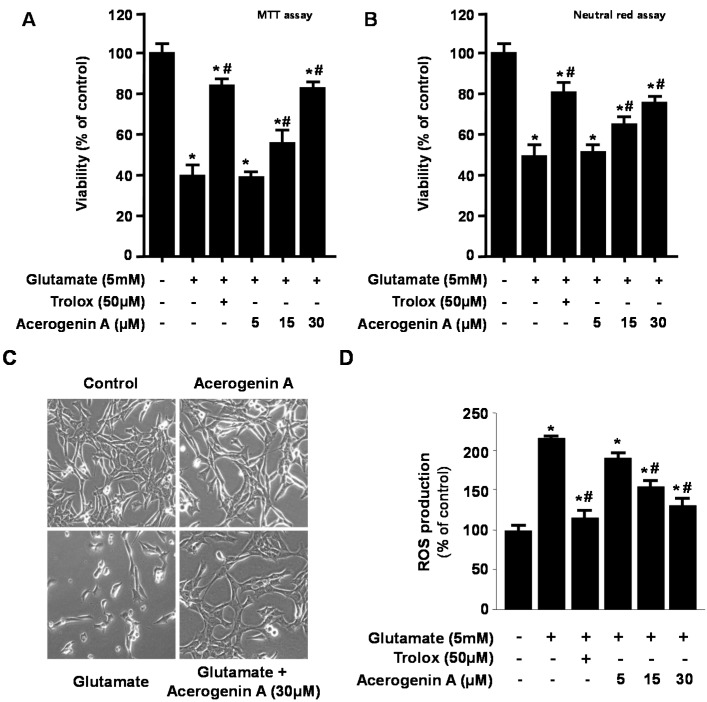
The effects of acerogenin A on glutamate-induced oxidative neurotoxicity (**A**–**C**) and inhibition of reactive oxygen species (ROS) generation (**D**). HT22 cells were pretreated with the indicated concentrations of acerogenin A for 12 h, and then treated with glutamate (5 mM) for 12 h (**A**–**C**). Exposure of HT22 cells to glutamate increased ROS production (**D**). Data are presented as mean ± SD values of three independent experiments. Trolox (50 μM) was used as the positive control. * *p* < 0.05 *vs*. control. **^#^** *p* < 0.05 *vs*. glutamate.

In this study, we examined the protective effects of acerogenin A against glutamate-induced cytotoxicity in mouse hippocampal HT22 cells. The MTT assay is a widely accepted cytotoxicity assay which can produce inaccurate results due to possible interference with the antioxidant property of phenolic compounds such as acerogenin A [16,17]. Therefore, we have also provided another evidence for the cytotoxic effects of acerogenin A by neutral red assay. To determine the cytotoxic potential of acerogenin A (Figure 1A), its effects on viability of HT22 cells (Figure 1B,C) were evaluated. Concentration of 30 μM revealed no cytotoxic effects using the MTT assay (Figure 1B) and neutral red assay (Figure 1C). However, a higher concentration at 40 μM showed a slightly reduced viability of these cells (Data not shown). Glutamate toxicity is a major contributor to pathological cell death within the nervous system [18]. Next, we investigated whether acerogenin A affected glutamate-induced oxidative neurotoxicity and ROS generation in HT22 cells. Next, we investigated to determine the protective effects of acerogenin A using the MTT assay (A) and neutral red assay (B). The viability of only glutamate-treated HT22 cells for 12 h was lower than in the control group, but pre-treatment with acerogenin A (5, 15 and 30 μM) for 6 h increased viability in a dose-dependent manner (Figure 2A–C). Glutamate also doubled ROS production, and acerogenin A effectively suppressed this induction (Figure 2D). Trolox, a well-known anti-oxidative agent [19,20], was used as positive control, and showed a significantly cytoprotective effect and ROS scavenging activity at a concentration of 50 μM.

### 2.2. Effects of Acerogenin A on Glutamate-Induced Oxidative Neurotoxicity through HO-1 Expression Pathway in HT22 Cells

**Figure 3:**
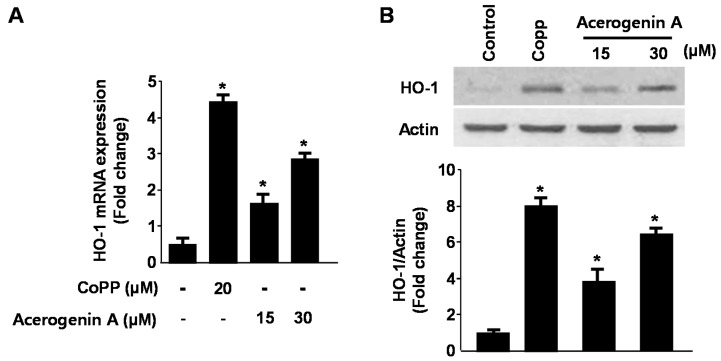

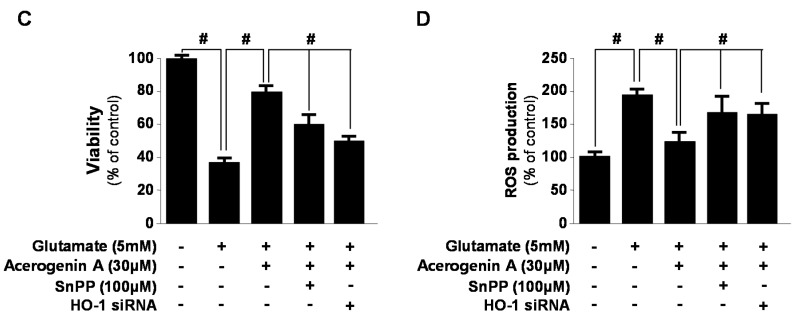
The effects of acerogenin A on heme oxygenase (HO)-1 expression (**A**,**B**) and acerogenin A-induced HO-1 on glutamate-induced oxidative neurotoxicity (**C**) and ROS generation (**D**). HT22 cells were incubated with indicated concentrations of acerogenin A for 12 h (**A**,**B**). HT22 cells were treated with 30 μM of acerogenin A in the presence or absence of 50 μM SnPP IX and HO-1 siRNA, and then exposed to glutamate (5 mM) for 12 h (**C**,**D**). Exposure of HT22 cells to 5 mM glutamate for 12 h to increase ROS production, followed by incubation with 10 μM of the ROS-sensitive fluorophore dichlorofluorescein (DCF) (**D**). Western blot analysis was performed, and representative blots of three independent experiments are shown. Data are presented as the mean ± SD values of three independent experiments. * *p* < 0.05 *vs*. control. ^#^ *p* < 0.05.

In our previous studies, we have demonstrated that HO-1 expression appeared to play key roles in cytoprotection in HT22 cells [19,20]. In addition, HO-1 and the subsequent metabolites of heme catabolism appear to play vital roles in regulating important biological responses including inflammation, oxidative stress, cell survival, and cell proliferation [21,22]. Therefore, we have provided evidence for the induction of HO-1 expression by acerogenin A in HT22 cells. At non-cytotoxic concentrations (15 and 30 μM), we examined whether acerogenin A affected HO-1 protein immunocontents by treating the HT22 cells with this agent for 12 h. Acerogenin A increased HO-1 mRNA expression in HT22 cells (Figure 3A). In addition, acerogenin A also dose-dependently increased HO-1 protein expression in HT22 cells (Figure 3B). CoPP (20 μM), a well-known HO-1 inducer, was used as the positive control [19,20]. To confirm that pre-incubation with acerogenin A markedly showed cytoprotective effect and ROS scavenging activity (Figure 1) and this effect was correlated with acerogenin A-mediated HO-1 expression (Figure 3), we investigated whether the effect of acerogenin A-mediated HO-1 expression was reversed by pre-treatment with SnPP, an inhibitor of HO-1 (Figure 3C,D) [19,20]. HT22 cells were co-treated with acerogenin A for 12 h in the absence or presence of SnPP, which is an inhibitor of HO-1 activity. SnPP significantly inhibited the acerogenin A-mediated cytoprotection using the MTT assay (Figure 3C). The acerogenin A-induced HO-1 expression was also required for suppressing glutamate-induced ROS generation (Figure 3D). In addition, the role of HO-1 expression by acerogenin A on the cytoprotective effect and ROS scavenging activity were studied using siRNA against HO-1. HT22 cells were transiently transfected with siRNA HO-1, and then were treated with acerogenin A followed by glutamate stimulation. As shown in Figure 3, SnPP treatment and transient transfection with HO-1 siRNA partially reversed the cytoprotective effect and ROS scavenging activity of acerogenin A. The results supported the hypothesis that induction of HO-1 contributes to the cytoprotective effects of acerogenin A on the HT22 cells.

### 2.3. Effects of Acerogenin A on Nrf2 Nuclear Translocation and Nrf2-Mediated HO-1 Expression in HT22 Cells

**Figure 4:**
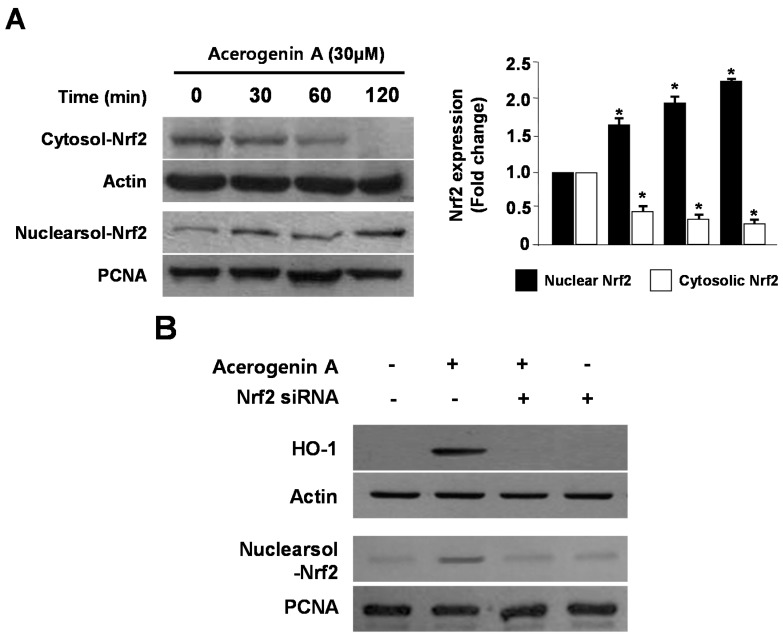
The effects of acerogenin A on the nuclear translocation of Nrf2 (**A**) and Nrf2-mediated HO-1 (**B**) in HT22 cells. HT22 cells were treated with 30 μM of acerogenin A for 0, 30, 60, and 90 min (**A**). The nuclei were fractionated from the cytosol by using PER-Mammalian Protein Extraction Buffer, as described in the Experimental Section (**A**). HT22 cells were transiently transfected with Nrf2 siRNA and then treated with 30 μM acerogenin A for 12 h (**B**). Western blot analysis was performed, and representative blots of three independent experiments are shown. Data are presented as the mean value ± SD values of three independent experiments. * *p* < 0.05 *vs*. control.

Numerous transcription factors participate in the regulation of HO-1 expression, with tendencies for one or more signaling pathways to predominate in a species-specific manner. Nrf2 is a basic leucine zipper transcription factor that resides in the cytoplasm bound to its inhibitor protein, Keap 1, and translocates to the nucleus after stimulation. It then binds to the ARE sequences in the promoter regions of specific genes [23]. Nrf2 has been known to induce the expression of antioxidant stress proteins such as HO-1 and glutathione (GSH) [24]. Therefore, we investigated whether treatment with acerogenin A induces the translocation of Nrf2 to the nuclei in HT22 cells. Cells were treated with acerogenin A for 30, 60, and 120 min, and the level of Nrf2 protein was then determined by western blotting. Western blot analysis of the nuclear fraction of acerogenin A-treated HT22 cells showed a gradual increase in Nrf2 levels, whereas a concomitant decrease was observed in the cytoplasmic fractions (Figure 4A). Furthermore, the role of Nrf2 in HO-1 expression by acerogenin A was studied using siRNA against Nrf2. HT22 cells were transiently transfected with siRNA Nrf2 and then were treated with acerogenin A for 12 h to induce HO-1 expression. As shown in Figure 4B, Nrf2 siRNA have completely blocked off nuclear translocation of Nrf2. In addition, transient transfection with Nrf2 siRNA also abolishes induction of HO-1 expression by acerogenin A in both HT22 cells. These results indicated that HO-1 induction upon incubation with acerogenin A is related to the Nrf nuclear translocation pathway in HT22 cells.

### 2.4. Involvement of the PI3K/Akt Pathway in Acerogenin A-Induced HO-1 Expression

**Figure 5:**
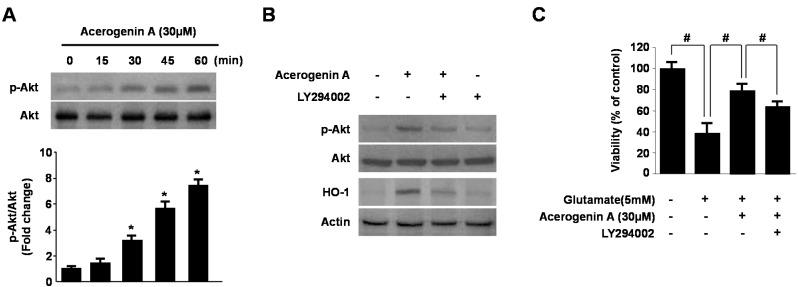
Effects of acerogenin A-induced HO-1 expression through the phosphatidylinositol 3-kinase (PI3K)/AKT cascade. HT22 cells were treated with acerogenin A (30 μM) for the indicated times (**A**). HT22 cells were pre-incubated with or without 10 μM LY294002 for 1 h and then incubated in the absence or presence of 20 μM of acerogenin A for 60 min (p-AKT) or 12 h (HO-1) (**B**). HT22 cells untreated or treated with acerogenin A (30 μM) in the presence or absence of LY294002 (10 μM) for 12 h were exposed to 5 mM glutamate for 12 h (**C**). Western blot analysis was performed, and representative blots of three independent experiments are shown. Data are presented as the mean ± SD values of three independent experiments. * *p* < 0.05 *vs*. control. ^#^ *p* < 0.05.

In previous studies, phosphatidylinositol 3-kinase (PI3K) was demonstrated to be involved in HO-1 induction by various phytochemicals [25]. Upstream signaling pathways were also implicated in the regulation of HO-1 expression, such as PI3K/AKT pathway [26]. Therefore, we tested whether acerogenin A-induced expression of HO-1 occurs through the PI3K pathway. To correlate the activation of Akt with the induction of HO-1 by acerogenin A, we investigated Akt phosphorylation by acerogenin A in HT22 cells by using an anti-phospho-Akt antibody. Akt was phosphorylated from 0 to 60 min by acerogenin A (Figure 5A), and its phosphorylation slowly declined thereafter in HT22 cells (Data not shown). Moreover, the PI3K pathway inhibitor (LY294002) abolishes acerogenin A-induced HO-1 expression (Figure 5B) and cytoprotection (Figure 5C) in HT22 cells. Therefore, we suggest that Nrf2-mediated HO-1 induction by acerogenin A is related to the PI3K/Akt pathway in HT22 cells.

## 3. Experimental Section

### 3.1. Chemicals and Reagents

Dulbecco’s modified Eagle’s medium (DMEM), fetal bovine serum (FBS), and other tissue culture reagents were purchased from Gibco BRL Co (Carlsbad, CA, USA). Cobalt protoporphyrin (CoPP), an inducer of HO-1, and tin protoporphyrin IX (SnPP IX), an inhibitor of HO activity, were obtained from Porphyrin Products. Lipofectamine 2000TM was purchased from Invitrogen Life Technologies (Grand Island, NY, USA). Small interfering RNA (siRNA) for HO-1 and Nrf2, and all other chemicals were obtained from Sigma Chemical Co. (St. Louis, MO, USA) unless otherwise stated. Primary antibodies, including mouse/goat/rabbit anti-HO-1, Nrf-2, p-Akt, Akt, and Actin, and secondary antibodies were purchased from Santa Cruz Biotechnology (Santa Cruz, CA, USA). Acerogein A was provided from Prof. Byung-Yoon Cha.

### 3.2. Cell Culture

Mouse hippocampal HT22 cells were received from Dr. Inhee-Mook (Seoul National University, Seoul, Korea). The cells were maintained at 5 × 10^6^ cells/dish in 100-mm dishes in DMEM supplemented with 10% heat-inactivated FBS, penicillin G (100 units/mL), streptomycin (100 μg/mL), and l-glutamine (2 mM), and incubated at 37 °C in a humidified atmosphere containing 5% CO_2_ and 95% air. For determination of cell viability, cells (2 × 10^4^ cells/well in 96-well plates) were incubated with 3-(4,5-dimethylthiazol-2-yl)-2,5-diphenyltetrazolium bromide (MTT) at a final concentration of 0.5 mg/mL for 4 h, and the formazan formed was dissolved in acidic 2-propanol. Optical density was measured at 590 nm with a microplate reader (Bio-Rad, Hercules, CA, USA). The optical density of the formazan formed in control (untreated) cells was considered to represent 100% viability.

### 3.3. MTT Assay and Neutral Red Assay

For determination of cell viability by MTT assay, HT22 cells (2 × 10^4^ cells/well in 96-well plates) were treated with acerogenin A in the presence or absence of 5 mM glutamate and incubated for indicated times. Next, the cell culture media from each well was removed, and the wells were rinsed multiple times with DPBS. After then, each wells replaced with 200 μL of fresh media. Next, cells were incubated with 3-(4,5-dimethylthiazol-2-yl)-2,5-diphenyltetrazolium bromide (MTT) at a final concentration of 0.5 mg/mL for 4 h, and the formazan formed was dissolved in acidic 2-propanol. Optical density was measured at 590 nm with a microplate reader (Bio-Rad, Hercules, CA, USA). The optical density of the formazan formed in control (untreated) cells was considered to represent 100% viability. In addition, cell viability in terms of lysosomal integrity was measured by neutral red assay (Sigma-Aldrich, New York, NY, USA). HT22 cells (2 × 10^4^ cells/well in 96-well plates) were treated with acerogenin A. Next, the cell culture media from each well was removed, and washed twice with PBS. After each time point, the media was removed, and the wells were rinsed multiple times with DPBS. After the DPBS washes, 200 μL of fresh media was added to each well along with 10 μL of neutral red reagent (0.33% in DPBS), followed by incubation for 4 h at 37 °C. The neutral red reagent was removed, and cells were treated with 100 μL of neutral red assay fixative for 1 min followed by 100 μL of neutral red assay solubilization reagent. Absorbance of culture media at 490 nm was used for microplate reader (Bio-Rad, Hercules, CA, USA). The optical density formed in control (untreated) cells was considered to represent 100% viability.

### 3.4. ROS Measurement

For measurement of ROS, HT22 cells (2.5 × 10^4^ cells/well in 24-well plates) were treated with 5 mM glutamate in the presence or absence of acerogenin A or SnPP IX (HO inhibitor) and incubated for 8 h. After washing with phosphate-buffered saline (PBS), the cells were stained with 10 μM 2′,7′-dichlorofluorescein diacetate (DCFDA) in Hank’s balanced salt solution for 30 min in the dark. The cells were then washed twice with PBS and extracted with 1% Triton X-100 in PBS for 10 min at 37 °C. Fluorescence was recorded at an excitation wavelength of 490 nm and an emission wavelength of 525 nm (Spectramax Gemini XS; Molecular Devices, Sunnyvale, CA, USA). Cells were immediately observed under a laser-scanning confocal microscope (Leica TCS SP2). DCF fluorescence was excited at 488 nm with an argon laser, and the evoked emission was filtered with a 515-nm long pass filter.

### 3.5. Preparation of Nuclear and Cytosolic Fractions

Cells were homogenized (1:20, *w*/*v*) in PER-Mammalian Protein Extraction buffer (Pierce Biotechnology, Rockford, IL, USA) containing freshly added protease inhibitor cocktail I (EMD Biosciences, San Diego, CA, USA) and 1 mM phenylmethylsulfonyl fluoride. The cytosolic fraction of cells was prepared by centrifugation at 15,000*×* *g* for 10 min at 4 °C. Nuclear and cytoplasmic extracts of HT22 cells were prepared using NE-PER nuclear and cytoplasmic extraction reagents (Pierce Biotechnology, Rockford, IL, USA), respectively. After treatment, HT22 cells (3 × 10^6^ cells/3 mL in a 60-mm dish) were collected and washed with PBS. After centrifugation at 1000 rpm for 3 min, cell lysis was performed at 4 °C by vigorous shaking for 15 min in RIPA buffer (150 mM NaCl, 1% NP-40, 0.5% sodium deoxycholate, 0.1% sodium dodecyl sulfate, 50 mM Tris-HCl (pH 7.4), 50 mM glycerophosphate, 20 mM NaF, 20 mM ethylene glycol tetraacetic acid, 1 mM dithiothreitol, 1 mM Na_3_VO_4_, and protease inhibitors). After centrifugation at 14,800× *g* for 15 min, the supernatant was separated and stored at −70 °C until further use. The protein content was determined using a bicinchoninic acid protein assay kit.

### 3.6. Western Blot Analysis

Cells were harvested and pelleted by centrifugation at 200× *g* for 3 min. Subsequently, the cells were washed with PBS and lysed in 20 mM Tris-HCl buffer (pH 7.4) containing a protease inhibitor mixture (0.1 mM phenylmethylsulfonyl fluoride, 5 mg/mL aprotinin, 5 mg/mL pepstatin A, and 1 mg/mL chymostatin). The protein concentration was determined using the Lowry Protein Assay Kit (P5626; Sigma Chemical Co.). An equal amount of protein from each sample was resolved using 12% sodium dodecyl sulfate-polyacrylamide gel electrophoresis and then electrophoretically transferred onto a Hybond-enhanced chemiluminescence nitrocellulose membrane (Bio-Rad). The membrane was blocked with 5% skimmed milk and incubated with anti-HO-1, anti-Nrf2, anti-Akt, anti-phospho-Akt, or anti-actin antibodies (all of which were used at a 1:1000 dilution and purchased from Santa Cruz Biotechnology) at 4 °C overnight. The immunoreactive bands were visualized using a horseradish peroxidase-conjugated secondary antibody (1:1000 dilution; Santa Cruz Biotechnology) followed by enhanced chemiluminescence detection (Amersham Pharmacia Biotech, Piscataway, NJ, USA) and quantified using an image analysis program (Image Gauge v3.12 software; Fujifilm, Tokyo, Japan).

### 3.7. Transfection

Cells were transiently transfected with HO-1 siRNA and Nrf2 siRNA for 6 h by using Lipofectamine 2000™ (Invitrogen, Carlsbad, CA, USA) according to the manufacturer’s protocol, and recovered in fresh media containing 10% FBS for 24 h.

### 3.8. RNA Quantification

Total RNA was extracted from harvested cells for reverse transcription polymerase chain reaction (RT-PCR) using the TRIzol System (Invitrogen). The RNA isolation protocol included a DNase I treatment. We quantified RNA and reverse transcribed cDNAs from 1 μg of total RNA per 20 μL RT reaction with Oligo (dT) 12–8 primers and the SuperScript First-Strand Synthesis System for RT-PCR (Invitrogen, Carlsbad, CA, USA). RT-PCR was conducted in a 25 µL solution containing 67.7 mM Tris-HCl (pH 8.8); 16.6 mM (NH_4_)_2_SO_4_; 0.01% Tween-20; 200 nM each of dATP, dCTP, and dGTP; 400 nM dUTP; 4.5 mM MgCl_2_; 300 nM of each primer; 200 nM probe; 2 U Taq DNA polymerase and 1/10 (by volume) of the cDNA synthesis reaction. Thermal cycling conditions consisted of 4 min at 95 °C followed by 25 cycles of 15 s at 95 °C and 1 min at 60 °C. The primer sequences were as follows: HO-1, forward 5′-CTCTTGGCTGGCTTCCTT-3′, reverse 5′-GGCTCCTTCCTCCTTTCC-3′, and glyceraldehyde 3-phosphate dehydrogenase (GAPDH), forward 5′-ACTTTGGTATCGTGGAAGGACT-3′, reverse 5′-GTAGAGGCAGGGATGATGTTCT-3′.

### 3.9. Statistical Analysis

Data were expressed as the mean ± SD of at least three independent experiments. To compare three or more groups, one-way analysis of variance (ANOVA) was used followed by Newman–Keuls *post hoc* test. Statistical analysis was performed using GraphPad Prism software version 3.03 (GraphPad Software Inc., San Diego, CA, USA).

## 4. Conclusions

In conclusion, we demonstrated that acerogenin A effectively prevents glutamate-induced oxidative damage, and HO-1 induction via PI3K/Akt and Nrf2 pathways appears to play a key role in the protection of HT22 cells. This study provides evidence that acerogenin A can exert a neuroprotective effect via activation of the Nrf2-mediated HO-1 expression. To our knowledge, the present study is the first to show that natural compound, acerogenin A, activates Nrf2-mediated HO-1 signaling in HT22 cells and exerts an anti-oxidative defense mechanism against glutamate-induced neurotoxicity. Therefore, this study implies that the Nrf2/HO-1 pathway represents a biological target and that acerogenin A might be a candidate for the prevention of neurodegeneration.
